# Topical treatment of vulvodynia, dyspareunia and pudendal neuralgia: A single clinic audit of amitriptyline and oestriol in organogel

**DOI:** 10.1111/ajo.13292

**Published:** 2021-01-11

**Authors:** Chantelle M. Ruoss, Elizabeth A. Howard, Karen Chan, Paul G. Stevenson, Thierry Vancaillie

**Affiliations:** ^1^ Women’s Health and Research Institute of Australia (WHRIA) Sydney New South Wales Australia; ^2^ Telethon Kids Institute Perth Children’s Hospital Perth Western Australia Australia

**Keywords:** dyspareunia, pelvic pain, pudendal neuralgia, vaginal mesh, vulvodynia

## Abstract

**Background:**

Vulvodynia and pudendal neuralgia comprise significant contributors to vulvar‐related pain and its impact on daily life.

**Aim:**

A retrospective clinical audit was conducted at the Women’s Health & Research Institute of Australia, Sydney, to determine the pattern of use and the efficacy of the application of topical amitriptyline 0.5% plus oestriol 0.03% in organogel (AOO), to the vulvar vestibule in reducing the impact of pain on daily life.

**Materials and Methods:**

There were 1174 patients who received a script from May 2017 until February 2020: 1054 patients agreed to be contacted and had a valid email address.

**Results:**

There were 376 (35.7%) patients who replied. Pain with intercourse was the main indication for use. Treatment was rated effective by 51.2% (95% CI: 35.4–66.8%) of patients less than 30 years of age, 66.7% (95% CI: 57.3–74.9%) of patients 30–50 years of age, and 58.3% (95% CI: 50.9–65.4%) in patients over 50. Stinging at the site of application was the most commonly reported side effect.

**Conclusion:**

Topical AOO is an effective and well‐tolerated treatment for vulvar pain.

## Introduction

The impact of vulvodynia and pudendal neuralgia can be significant on a woman’s quality of life, affecting her mental health, intimacy, and activities of daily living both socially and occupationally. Dyspareunia is a common and debilitating condition with a prevalence up to 39.5%. Vulvodynia has an estimated lifetime prevalence varying between 8% and 28%.^1^, ^2^, ^3^ Typical symptoms include burning, stinging, itching or stabbing; with the diagnosis being one of exclusion.^2^, ^3^, ^4^ Pudendal neuralgia, like vulvodynia, is a pain condition diagnosed clinically in the absence of other pathology.^5^ Characteristic symptoms include urinary frequency and urgency, dysuria, dyschezia, vaginal burning, dyspareunia, rectal or vaginal foreign body sensation and sensitivity to touch in the perineum.

Although the pudendal nerve does not innervate the bladder or bowel directly, neurogenic inflammation likely contributes to cross‐sensitisation resulting in the wide spectrum of symptomatology in both vulvodynia and pudendal neuralgia. Ongoing peripheral input at the shared sacral dorsal root ganglions results in release of neurotransmitters and peptides including substance P, calcitonin gene‐related peptide and nerve growth factor in adjacent pelvic structures.^6^

Management of vulvodynia and pudendal neuralgia should be individualised to each patient with current evidence supporting the role for oral antidepressants, anticonvulsants; topical preparations of anaesthetics, oestrogen, corticosteroids and pudendal nerve blocks. Non‐pharmacological treatment plays an equally important role and can comprise of physical therapy including nerve glides, pain education and lifestyle modifications such as loose clothing and standing desks.^3^, ^7^, ^8^

Targeted therapy with topical compounded ointments or creams has the benefit of reduced systemic side effects while concurrently avoiding interpatient variability with oral dosing regimens.^7^ Few studies have investigated the use of topical amitriptyline for the management of vulvodynia. Pagano *et al*. assessed the use of topical amitriptyline 2% in sorbolene cream with 33.9% of patients positively responding to treatment.^9^ In other studies, topical amitriptyline alone or in combination with other medications has been safely used in up to a concentration of 10%.^8^, ^9^, ^10^, ^11^, ^12^ Topical oestrogen has been shown to provide improvement in symptoms in postmenopausal women in a study by Goetsch *et al*.^13^ although it should be noted this was a small study. Treatment of long‐term oestrogen deficiency has also been shown to ameliorate bladder symptoms.^14^ Based upon evidence supporting the efficacy of individual amitriptyline and oestriol, the combination was devised as a possible improved means of treating vulvar‐related pain.

AOO consists of amitriptyline 0.5% and oestriol 0.03% in organogel. This amounts to 5 mg and 0.3 mg per mL of gel respectively. An organogel was chosen as the base, because of its amphiphilic properties. The recommended dose is 0.5 mL applied to the vulva once or twice a day. The maximum daily dose is therefore 5 mg of amitriptyline and 0.3 mg of oestriol. These present as low doses compared to topical or oral medications currently commercially available. The premise for its use focuses on symptomatic treatment of vulvo‐vaginal pain, irrespective of aetiology, through reduction of the sensitivity of the mucosa by the membrane stabilising effect of amitriptyline. Additionally, the stimulatory effect of oestriol promotes increased mucosal regeneration, flexibility and lubrication.^15^ This combination topical preparation has not previously been described in the literature. AOO is a proprietary formulation (Australian patent # 2017259104), which is available from a select number of compounding pharmacies under the trade name Mi‐Gel^®^ (Australian trademark # 1376821).

## Materials and Methods

Ethics approval to contact patients suitable for clinical audit was obtained (Bellberry Human Research & Ethics Committee approval number 2020‐02‐083). A search of our clinical records system identified patients who were issued a script for AOO from May 2017 to February 2020 by their treating physician. Of the 1174 females identified, 1127 individuals had previously consented to being contacted for research purposes. Of these, 1054 with valid email addresses were invited to complete the survey.

A single electronic survey to obtain demographic data and outcome measures including the validated Pelvic Pain Impact Questionnaire^16^, ^17^ was developed via Delphi process involving 22 individuals (nine physicians, seven allied health practitioners, six lay people). All responses were anonymous to protect patient privacy and they could opt‐out at any time via not completing the survey.

The χ^2^ test statistic was used to compare categorical data with statistical significance threshold of *P* < 0.05. Where the null hypothesis was rejected, post hoc comparisons were made by examining the standard residuals after adjusting the critical *z*‐score value per the Bonferroni method. Logistic regression was performed on dichotomous outcome (ie modelling responses of ‘Yes’ compared to ‘No’, responses of ‘Not sure’ were excluded from regression analysis). Ordinal logistic regression was used to model the association of side effects and duration of use.^18^ All regression analyses were adjusted for age and reason for use. All data wrangling and analysis were completed in the R programming language for statistical analysis.^19^ Missing data were excluded from analysis.

## Results

Women invited to participate in the audit ranged from 18 to 89 years of age. A total of 376 out of 1054 responded to the online survey (35.7% responder rate). Incomplete responses to individual questions were removed from data analysis resulting in variable total counts for each question. The age distribution of the survey respondents compared to the cohort was equivalent in the 30–50 group (*P* = 0.66) with a smaller representation of the less than 30 years old in the audit sample (18.3% vs 11.3%, *P* = 0.002), with an equivalent greater representation of the over 50 years old group (46.2% vs 54.7%, *P* = 0.005). Of the 376 respondents, 47 (12.5%) reported vaginal mesh problems, which is statistically consistent with the proportion in the total cohort with mesh (132/1161 = 11.4%, *P* = 0.62).

Pain with intercourse was the main indication for use across all ages (206/376 = 54.8%) followed by pain with sitting (124/376 = 33.0%), bladder issues (76/376 = 20.2%) and vaginal mesh problems (47/376 = 12.5%). Some patients mentioned more than one reason for use of AOO. Women self‐reported AOO to be beneficial across all age groups (see Table 1), with 51.2% (95% CI: 35.4–66.8%) of the 30 years or less age group; 66.7% (95% CI: 57.3–74.9%) of 30 to 50‐year‐old group, and 58.3% (95% CI: 50.9–65.4%) of the 50 or older age group. When comparing reasons for use against overall outcome, 67.2% of those with pain on sitting reported the gel to be efficacious, followed closely by those with bladder issues (67.1%). AOO appears to be equally effective in reducing each symptom (Table 2).

Respondents were asked to indicate their duration of use ranging from short term use (up to 3 months), to ongoing (>12 months) use (Table 3). The indication with greatest ongoing use beyond 12 months was bladder issues (45.1%).

**Table 1:** Overall efficacy of topical amitriptyline 0.5% plus oestriol 0.03% in organogel (partial respondents excluded thereby altering sum total to 345)

Response	Younger than 30	30–50	50 or older
Yes	21 (51.2%)	78 (66.7%)	109 (58.3%)
No	8 (19.5%)	18 (15.4%)	37 (19.8%)
Not sure	12 (29.3%)	21 (17.9%)	41 (21.9%)
Total	41	117	187

**Table 2:** Relationship between reason for use and outcome

Reason	Yes	No	Not sure	Total
Bladder issues	51 (67.1%)	11 (14.5%)	14 (18.4%)	76
Pain with intercourse	132 (64.4%)	33 (16.1%)	40 (19.5%)	205
Pain with sitting	82 (66.7%)	22 (17.9%)	19 (15.4%)	123
Vaginal mesh problems	22 (46.8%)	11 (23.4%)	14 (29.8%)	47

There is no evidence (χ^2^ test, *P* = 0.26) to reject the null hypothesis that the relative proportions of outcomes are different between topical amitriptyline 0.5% plus oestriol 0.03% in organogel reasons for use.

**Table 3:** Relationship between reason for use and duration of use

Reason	≤1 month	1–3 months	3–6 months	6–12 months	>12 months	Total
Bladder issues	4 (5.6%)	15 (21.1%)	9 (12.7%)	11 (15.5%)	32 (45.1%)	71
Pain with intercourse	11 (5.6%)	61 (31.0%)	39 (19.8%)	34 (17.3%)	52 (26.4%)	197
Pain with sitting	6 (5.1%)	25 (21.4%)	27 (23.1%)	13 (11.1%)	46 (39.3%)	117
Vaginal mesh problems	3 (7.3%)	5 (12.2%)	14 (34.1%)	7 (17.1%)	12 (29.3%)	41

Counts of responses to each group paring and row percentages of those that responded to this question. There is evidence (χ^2^, *P* = 0.030) to reject the null hypothesis in favour of the alternative that at least one of the relative proportions of topical amitriptyline 0.5% plus oestriol 0.03% in organogel reasons for use between durations is different than what is expected.

Women aged 50 years or older were, on average, 2.21 times more likely to use the topical medication for longer than women in the under 30 age group (95% CI: 1.32–3.70), keeping constant the reason for use.

Ninety of the 376 (24.0%) patients reported one or more side effects (Table 4). Stinging at the application site for less than 10 min duration was the most common side effect in the groups utilising AOO for pain with intercourse, pain with sitting and bladder issues (16.1%, 14.8% and 12.7% respectively). In those utilising the gel for vaginal mesh problems, constipation was the most commonly recorded side effect (11.7%). Reporting of one or more side effects was lowest in the 50 or older age group (50/206, 24.3%), followed by the 30 to 50 age group (32/130, 24.6%) and those 30 years old or less (11/42, 26.2%).

**Table 4:** Relationship between reason for use and side effects

Side effect	Vaginal mesh problems	Pain with intercourse	Pain with sitting	Bladder issues
Stinging at application site, less than 10 min	6 (9.0%)	26 (16.8%)	18 (15.5%)	14 (13.9%)
Temporary itching at application site, less than 10 min	7 (10.4%)	17 (11.0%)	10 (8.6%)	10 (9.9%)
Persistent itching, needed to wash it off	1 (1.5%)	9 (5.8%)	4 (3.4%)	3 (3.0%)
Temporary burning at application site, less than 10 min	5 (7.5%)	18 (11.6%)	14 (12.1%)	10 (9.9%)
Persistent burning, needed to wash it off	3 (4.5%)	4 (2.6%)	5 (4.3%)	4 (4.0%)
Recurrent thrush	3 (4.5%)	13 (8.4%)	8 (6.9%)	7 (6.9%)
Local irritation, redness, rash, possible local allergy	3 (4.5%)	12 (7.7%)	8 (6.9%)	8 (7.9%)
Drowsiness	6 (9.0%)	8 (5.2%)	7 (6.0%)	5 (5.0%)
Dizziness	4 (6.0%)	7 (4.5%)	5 (4.3%)	4 (4.0%)
Headache	5 (7.5%)	6 (3.9%)	4 (3.4%)	4 (4.0%)
Constipation	6 (9.0%)	9 (5.8%)	10 (8.6%)	10 (9.9%)
Difficulty emptying bladder	7 (10.4%)	9 (5.8%)	9 (7.8%)	11 (10.9%)
Breast tenderness	6 (9.0%)	9 (5.8%)	10 (8.6%)	6 (5.9%)
Hives	1 (1.5%)	2 (1.3%)	1 (0.9%)	1 (1.0%)
Shortness of breath	4 (6.0%)	6 (3.9%)	3 (2.6%)	4 (4.0%)
Total	67	155	116	101

Counts of responses to each group paring and column percentages. With a *P*‐value of 1 (χ^2^ test), there is no evidence to reject the null hypothesis that there is a difference to the relative proportions in the number of reported side effects between topical amitriptyline 0.5% plus oestriol 0.03% in organogel reasons for use.

Reversibility was noted in 53% of patients with resolution of side effects on cessation of AOO.

## Discussion

Within the limitations of a retrospective audit, the results indicate that AOO is beneficial in the treatment of pudendal neuralgia type symptoms related to a variety of aetiologies.

The lower response rate to the audit in the younger age group is a commonly observed phenomenon. In a study conducted by Eaker *et al*.,^20^ assessing response rates to mailed questionnaires, a marked reduction in response rate was noted in those aged under 35 years. This trend was also noted in another study by Mannetje *et al*.^21^ where younger age was a strong predictor of non‐response. In this study, those in the younger age groups were two times less likely to participate. With equivalences in the middle age group and incidence of mesh neuropathy, we believe that the audit sample is representative of the cohort.

There were 64.4% of respondents who reported an improvement in vulvar pain during intercourse, also known as entry dyspareunia. This positive response compares favourably with the use of other topical preparations. Pagano *et al*., for instance, noted a 34–56% positive response rate when using amitriptyline 2% in sorbolene cream for the management of patients with entry dyspareunia; 10% of these patients reported complete resolution of pain.^9^ It is interesting to note that urinary symptoms, such as urgency, also improve at a similar rate with topical application of AOO to the vulva. The presence of urinary symptoms was the reason for long‐term use of the gel with 44.6% continuing to use it for greater than 12 months duration.

Typical adverse effects of amitriptyline with oral administration most commonly include drowsiness, dizziness, dry mouth and constipation among other anticholinergic effects. Topical administration carries the benefit of increased local tissue uptake rather than systemic, theoretically reducing the side effect profile.^7^, ^22^ There is a relative paucity of evidence in the current literature detailing adverse effects of topical amitriptyline specifically for treatment of vulvo‐vaginal pain. A systematic review evaluating topical amitriptyline for neuropathic pain sited common adverse effects of skin irritation, itching, burning, sedation, dry mouth in addition to cardiac effects of rapid heart rate and palpitations. Of note, the topical preparations comprised of amitriptyline concentrations between 1% and 5%.^22^ The majority of side effects reported by 24% of the respondents from this audit were minor and resolved with cessation of application.

Based on the results of this audit, a prospective randomised placebo‐controlled study has been designed to further qualify the efficacy of AOO for the treatment of entry dyspareunia.
